# Constrictive bronchiolitis obliterans with a presumptive etiology of preceding feline herpesvirus infection in a cat

**DOI:** 10.1186/s12917-022-03368-4

**Published:** 2022-07-05

**Authors:** Pin-I Hsieh, Hui-Wen Chen, Hsiao-Ning Yeh, Man-Cham Lam, Pei-Ying Lo, Wei-Hsiang Huang, Cheng-Hsin Shih, Chung-Hui Lin

**Affiliations:** 1 Department of Veterinary Medicine, National Taiwan University, Taipei, Taiwan; 2 National Taiwan University Veterinary Hospital, National Taiwan University, Taipei, Taiwan; 3 Graduate Institute of Molecular and Comparative Pathobiology, Department of Veterinary Medicine, National Taiwan University, Taipei, Taiwan; 4 Graduate Institute of Veterinary Clinical Sciences, School of Veterinary Medicine, National Taiwan University, No. 1, Sec 4, Roosevelt Road, Taipei, Taiwan

**Keywords:** Bronchiolar disorders, Bronchoalveolar lavage, Cat, Constrictive bronchiolitis obliterans, Feline herpesvirus, Lung high-resolution computed tomography, Respiratory disease

## Abstract

**Background:**

Bronchiolar disorders are rarely recognized in cats. Constrictive bronchiolitis obliterans is characterized by concentric peribronchiolar fibrosis and inflammation of the bronchioles, but the underlying causes remain poorly understood in current small animal medicine.

**Case presentation:**

A 9-year-old cat presented with paroxysmal tachypnea, infrequent cough and persistent labor breathing. Thoracic radiography showed lung hyperinflation and bronchointerstitial pattern, and pulmonary function assessment revealed flow limitation in the late-expiratory phase and poor response to short-acting bronchodilator. Dorsally distributed subpleural ground glass opacities with distinct margin and tree-in-bud opacities were observed on lung high-resolution computed tomography. The cat underwent bronchoalveolar lavage (BAL) and showed severe neutrophilic inflammation. Feline herpesvirus was the only pathogen detected in the BAL fluid. Multiple therapeutic attempts were unsuccessful and the cat died 8 weeks after the initial presentation. Necropsy revealed the infiltration of inflammatory cells, obstruction of the bronchiolar lumen, and submucosal concentric fibrosis suggesting constrictive bronchiolitis obliterans. Combining the pre- and post-mortem findings, as well as the time from symptom onset or BAL to necropsy, constrictive bronchiolitis obliterans was possibly triggered by a preceding feline herpesvirus infection in this case.

**Conclusions:**

The history of nonvaccinated status, lower airway neutrophilic inflammation, and presence of feline herpesvirus in the BAL fluid without coexistence of other pathogens led to the presumption that constrictive bronchiolitis obliterans was induced by a preceding feline herpesvirus infection in this cat. The pathological changes of bronchiolitis obliterans induced by a preceding feline herpesvirus infection could be different from that of cats with acute herpesvirus pneumonia, such as intranuclear inclusions would disappear over time and were no longer found 7–10 days after inoculation. The presence of patchy distribution of subpleural ground glass opacities on lung high-resolution computed tomography should raise the suspicion of peribronchiolar fibrosis. Clinical awareness of bronchiolar disorders as a differential diagnosis is important in cats with lung hyperinflation and labored breathing who show poor reversibility to bronchodilator.

## Background

In cats, bronchiolar disorders are underinvestigated in veterinary clinical medicine. With increased application of high-resolution computed tomography (HRCT), bronchiolar abnormalities are being recognized more frequently than previously in small animal patients [1–3]. Bronchiolar disorders in human medicine are associated with a wide spectrum of etiologies, and the diagnosis is made in combination with clinical manifestation, functional characteristics, imaging findings, and histopathologic evidence [4–6]. However, premortem diagnosis is rarely reported in the current veterinary literature, and the underlying causes remain largely unknown.

Constrictive bronchiolitis obliterans is considered as one of the primary bronchiolar disorders and characterized by concentric peribronchiolar fibrosis and inflammation of the bronchioles [1]. Naturally occurred constrictive bronchiolitis obliterans has only been recently reported in a dog and shortly described in a cat to date [1, 2]. This case report describes the clinical course, thoracic radiographic and lung HRCT findings, pulmonary function assessment, bronchodilation test, and cytology as well as the presence of viral pathogen in bronchoalveolar lavage (BAL) fluid in an adult cat with constrictive bronchiolitis obliterans confirmed at necropsy. A preceding feline herpesvirus infection was speculated to play a key role in triggering the disease process.

## Case presentation

A 9-year-old, 3.5 kg, spayed female domestic shorthair cat was evaluated for a 1.5-month history of persistent labored breathing. Three months prior to the presentation, the cat developed paroxysmal tachypnea that gradually worsened and was treated with theophylline, bromhexine and cetirizine by the primary veterinarian. An improvement was observed in the tachypnea, but signs of increased breath effort persisted. The owner reported that the cat had a 1-year history of infrequent cough, occurring approximately twice a month. The cat had not been vaccinated for uncountable years and was housed entirely indoors. The deworming prophylaxis was not administered on a regular basis. According to the owner, there was no known exposure to any indoor pollutant or toxic substance.

**Fig. 1:**
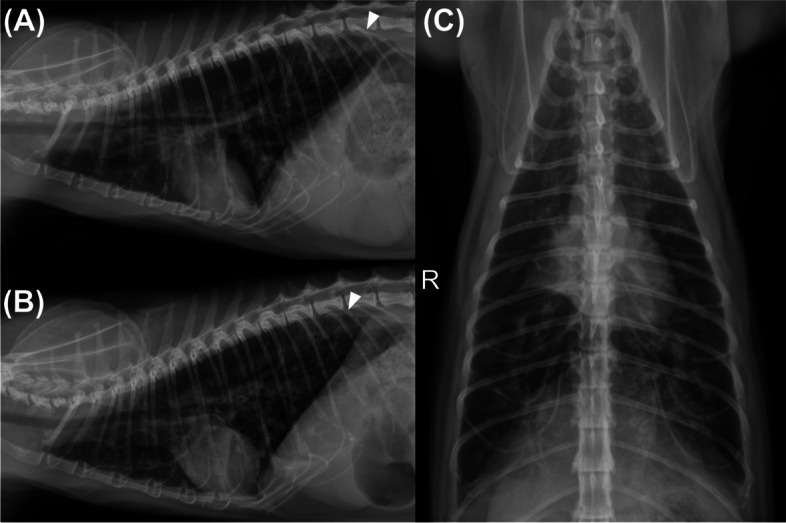
Right lateral (**A**), left lateral (**B**), and ventral-dorsal (**C**) radiographs of the thorax showing lung hyperinflation and generalized bronchointerstitial pattern with focally increased opacity in the right middle lung lobe and caudodorsal lung fields (white arrowhead)

At presentation, auscultation of the thorax revealed decreased lung sounds and subtle crackles. Hematology and serum biochemistry were unremarkable. Serological tests for *Toxoplasma gondii* IgG antibody, feline heartworm antibody, cryptococcus antigen, and FIV/FeLV antibody were negative. Thoracic radiography identified significant lung hyperinflation and diffuse bronchointerstitial pattern with focally increased opacity in the right middle lung lobe and caudodorsal lung fields (Fig. 1).

**Fig. 2:**
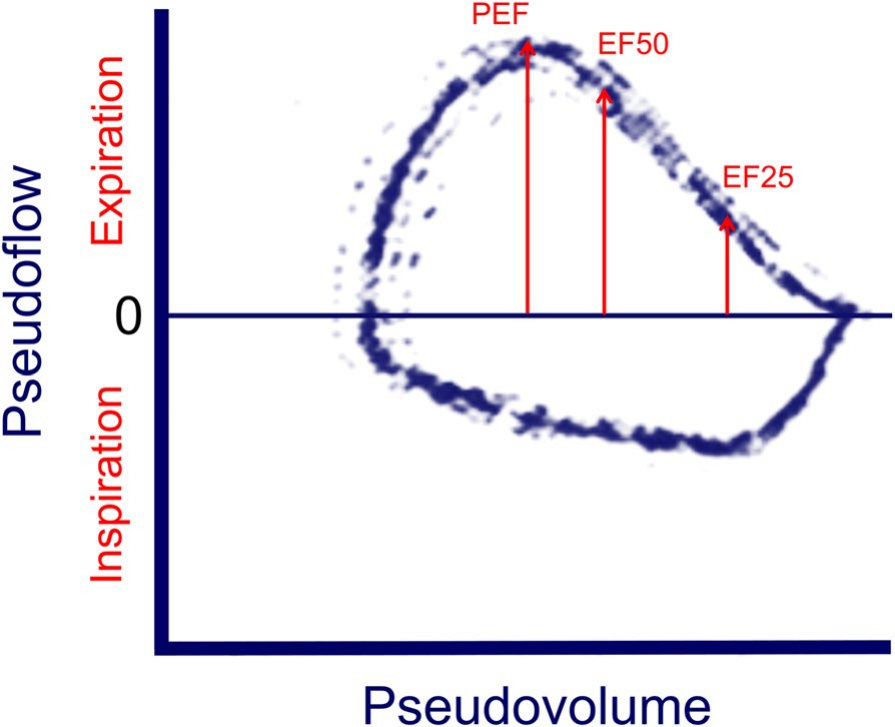
Pulmonary function assessment by pseudoflow and pseudovolume revealed flow limitation at the late-expiratory phase under natural tidal breathing. Abbreviations: PEF, peak expiratory flow; EF50 and EF25, expiratory flow at 50% and 25% of the remaining tidal volume during tidal breathing

Pulmonary function test was noninvasively performed using barometric whole-body plethysmography (BWBP), showing increased minute volume (906 mL/kg; in normal control cats, 299.8 ± 52.1 mL/kg) and late-expiratory flow limitation (pTBFVL PEF/EF25 ratio = 2.79; in normal control cats, 1.64 ± 0.18) under natural breathing (Fig. 2) [7]. Terbutaline (0.01 mg/kg) was administered subcutaneously as a bronchodilation test, as well as a treatment trial. After approximately 30 min, BWBP recording was repeated and revealed reduced minute volume (537 mL/kg; in normal control cats, 299.8 ± 52.1 mL/kg) and PEF/EF25 ratio (2.13; in normal control cats, 1.64 ± 0.18), which were still abnormally high, suggesting an incomplete response to the short-acting bronchodilator. The differential diagnosis at this stage was considered to be etiologies associated with lung hyperinflation and obstructive lower airway diseases, including feline lower airway disease (FLAD) with a phenotype of irreversible bronchoconstriction, emphysema, or lung bullae. Further diagnostics, such as lung HRCT, were suggested, and the owner preferred scanning under sedation alone to reduce the risk associated with anesthesia. Terbutaline (0.14 mg/kg PO q8h), prednisolone (0.6 mg/kg PO q12h), and enrofloxacin (2.5 mg/kg PO q12h) were prescribed as a trial for possible FLAD with concurrent lower respiratory tract infection.

**Fig. 3:**
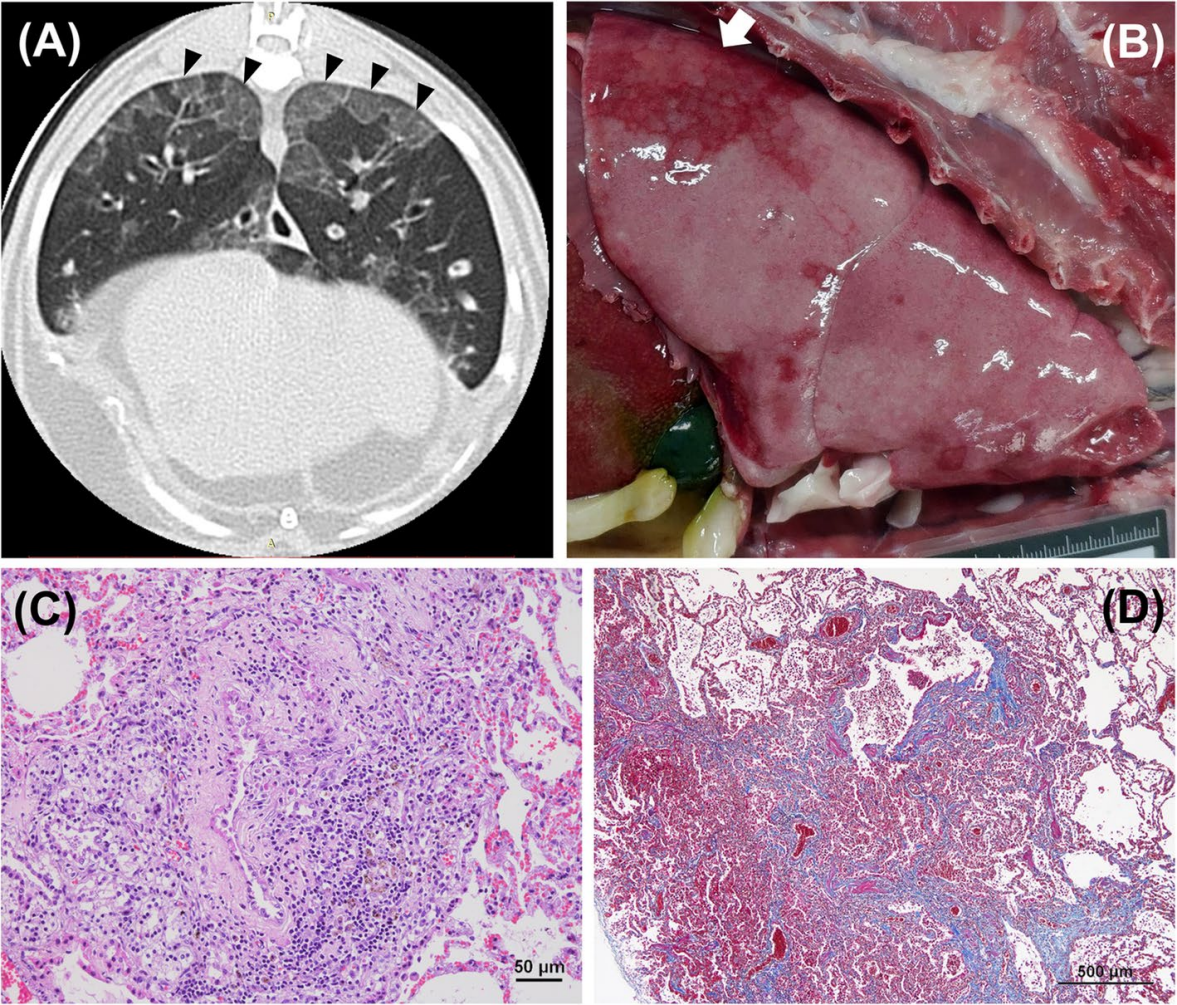
Subpleural ground-glass opacities with distinct margin were observed in the dorsal regions of the bilateral caudal lung lobes (black arrowhead) on lung high-resolution computed tomography (HRCT) (**A**). At necropsy, the dorsally distributed lesions on lung HRCT corresponded to the multifocal to coalescent dark-red foci (white arrow) on bilateral caudal lobes (**B**). Histopathology identified the fibrotic narrowing of the bronchioles (**C**) (Bar scale = 50 μm), and the alveolar septa around these bronchioles was thickened by moderate amount of fibrous connective tissue illustrated using Masson’s Trichrome staining (**D**) (Bar scale = 500 μm)

Lung HRCT was performed 2 days later revealing no evidence of emphysema or lung bullae. However, dorsally distributed and distinct marginated subpleural ground-glass opacities (GGO) were observed in the bilateral caudal lung lobes (Fig. 3A) and a small part of left cranial lobe. There seemed to have more severe bronchiectasis in the bronchi toward those regions. Other findings included tree-in-bud opacities and mildly thickened bronchial walls [8]. CT images did not support the tentative diagnosis of typical FLAD, and the suspicion of an infectious etiology was raised. Prednisolone was temporarily withdrawn in concern of the presence of an active infection, and clindamycin (12 mg/kg PO q12h) was prescribed additionally.

After a 10-day course of antibiotics and bronchodilator treatment, the cat experienced an improvement in activity levels at home but still showed labored breathing, which seemed to become worse gradually. The owner agreed to the BAL procedure at this time point, and the sampling was scheduled in 6 days with immediate withdrawal of antibiotics. Nonbronchoscopic BAL was performed by aseptically passing a sterile 8-Fr polyvinylchloride tube through the endotracheal tube to be wedged in the distal airway. Dorsal recumbency was used in this cat to obtain samples from the dorsal region of the lung, which had multiple subpleural GGO on CT images. Two boluses of 7.5-mL warmed sterile saline (followed by 2-mL air) were instilled, and the recovered fluid (8.2 mL) was turbid and had substantial amounts of mucus. Only one lung was sampled to avoid compromising the patient’s condition further. BAL fluid was processed immediately after collection, and cytology showed hypercellularity (1037 cells/μL; reference interval [RI] 200–400/μL) with 88.5% neutrophils (RI < 7%), 7.7% macrophages (RI 65%–80%), 3.4% lymphocytes (RI < 10%), and 0.4% eosinophils (RI < 17%) [9–11]. Routine microbiological examination, including aerobic and anaerobic bacterial culture, *Mycoplasma* PCR, and fungal culture were all negative. Additional workup was attempted to investigate other common viral pathogens by PCR, including feline coronavirus, calicivirus, and herpesvirus; the first two were negative, and the latter was positive in the BAL sample.

Considering the presence of herpesvirus in the BAL fluid, as well as negative results of other pathogens, the lack of adequate vaccination in this cat, and other clinical findings, herpesvirus-induced bronchiolitis was diagnosed accordingly. Owing to no known standard treatment for viral bronchiolitis in small animal medicine, the therapeutic strategy was focused on supportive care, bronchodilation, mucolytics, and anti-inflammatory treatment. In the subsequent 6 weeks, multiple attempts of different bronchodilator (terbutaline, PO or SC; sustained-released theophylline, PO; ipratropium, nebulized) were used separately or in combination; none of these could effectively alleviate the labored breathing of the cat. Inhaled form of corticosteroids was considered, but the owner failed to achieve cooperation from the cat. Oral prednisolone was prescribed at a relatively low anti-inflammatory dose of 1.1 mg/kg/day, with careful observation for possible reactivation of the virus. Previously prescribed enrofloxacin was continued with the aim of preventing secondary bacterial infection, and it was later replaced with doxycycline for its potential immunomodulating effect. Lysine was supplemented at a dose of 1000 mg/day, and no other antiviral agent was considered due to the absence of strong evidence for treatment efficacy. The activity, appetite, and breathing effort of the cat waxed and waned in the 5 weeks after BAL, but the overall clinical status deteriorated rapidly over the last few days. The cat died 8 weeks after the initial presentation.

Necropsy showed a hyperinflated lung with geographic, dark-red, well-demarcated, and slightly firm foci on bilateral caudal lobes and the left cranial lobe (Fig. 3B). There were numerous small air bubbles in the pulmonary parenchyma of every lobe which is suggestive of overinflation of alveoli. Excessive yellow-green mucopurulent exudate filled the bilateral bronchi. Histopathology identified that most lesions were centered on the bronchioles, with extension into the interstitium and subpleural distribution. Some bronchi were also affected, and both the bronchial and the bronchiolar lumina were filled with abundant mucus, amorphous cell debris, neutrophils and macrophages, with epithelial squamous metaplasia. Submucosal gland hyperplasia with moderate to marked lymphoplasmacytic inflammation was also present. There was prominent bronchiolar epithelial hyperplasia with severely hypertrophic smooth muscles surrounding the bronchi and bronchioles, and the adjacent alveolar septa revealed moderate interstitial fibrosis (Fig. 3C). The small bronchioles exhibited a varying degree of submucosal concentric fibrosis compressing and reducing the diameter of the lumen, with lymphoplasmacytic infiltrates (Fig. 3D). These histologic changes were consistent with constrictive bronchiolitis obliterans [2, 12, 13]. Within the remaining aerated lung, the alveolar spaces were enlarged and coalescent. However, typical pathognomonic lesions associated with feline herpesvirus infection, such as tissue necrosis, syncytial cells, and viral inclusion bodies, were not found in the histopathological examination. A piece of lung tissue from the dorsal region of the caudal lung lobe was collected and submitted for feline herpesvirus PCR, but the virus was no longer detected. Considering the time from symptom onset or BAL to necropsy, it was speculated that the pathological changes could be different from that of cats with acute herpesvirus pneumonia. Therefore, constrictive bronchiolitis obliterans induced by a preceding feline herpesvirus infection remains highly suspected in this case.

## Discussion and conclusions

Bronchiolar disorders in cats have not been extensively recognized in small animal medicine, and bronchiolitis has been rarely reported as the primary clinical presentation presumptively caused by common feline herpesvirus. Most cats diagnosed with or suspected to have bronchiolar disease in the literature are older in age and have no breed predisposition [1], as seen in our case. In contrast, dogs diagnosed with bronchiolar disease were purebred and young although only few cases have been described [2, 3]. In humans, bronchiolar disease can be diagnosed in any age group; postinfectious bronchiolitis is more common in young children, whereas aspiration bronchiolitis is more common in the elderly with oropharyngeal dysphagia [6, 14]. The age at diagnosis is likely associated with the underlying etiology, and the prevalent triggers or risk factors of this disease in cats remain unclear.

Labored breathing is a common clinical sign present in both veterinary and human patients with bronchiolar disease [1–3, 13]. Although cats with asthma could also experience labored breathing caused by bronchoconstriction, the increase in breathing effort is typically episodic, self-limiting, or responds well to short-acting bronchodilator [15]. None of these classic characteristics of feline asthma were observed in our patient, leading to the suspicion of an uncommon disease. Chronic cough is another common clinical sign found in cats and dogs with bronchiolar disease but indistinguishable from other lower airway diseases [1–3, 16]. Signalment and physical examination findings, such as crackles and wheezing, are not specific to bronchiolar disease either in the current case or in previous reports, but the presence of persistent labored breathing and a poor response to bronchodilators should increase the suspicion of bronchiolar disease.

Thoracic radiographs in our cat did not provide many clues for considering any uncommon differential diagnoses at the beginning, except for lung hyperinflation, implying the presence of lower airway obstruction, emphysema, or bullae [1, 17, 18]. In previously described case series, radiographic features in cats with bronchiolar disease were variable, including mixed or unstructured interstitial/alveolar pattern, peribronchial infiltrates, hyperinflated lung, atelectasis, or consolidation [1, 19]. Although it has been reported that thoracic radiography could even be normal in human patients with bronchiolar disease [6, 13], all feline patients with bronchiolar disease seemed to show certain significant abnormalities on thoracic radiographs [1, 19]. In our case, a hyperinflated lung along with inappropriate reversibility after short-acting bronchodilator use led to the application of lung HRCT, which revealed abnormalities related to bronchiolar disease, including multifocal peribronchiolar GGO and tree-in-bud signs [1, 8, 19, 20]. Lung HRCT scanning is the most valuable diagnostic tool for bronchiolar disorders in human patients, and recognizing various HRCT patterns could assist in the differential diagnosis [6]. Notably, terms such as “centrilobular” used for describing lesion patterns in human medicine cannot be applied to our patient because of the anatomical differences among species and the lack of secondary pulmonary lobules in cats [1, 6]. However, little is known about the HRCT imaging characteristics in each entity of the recently proposed classification scheme for feline bronchiolar disorders [1, 19]. The patchy distribution of peribronchiolar GGO around the dorsal aspect of the caudal lung lobes was found to be bronchiolocentric fibrosis on necropsy in our cat. Therefore, the presence of such HRCT patterns in future cases might suggest the development of constrictive bronchiolitis obliterans, and histopathologic evaluation should be considered whenever applicable.

Pulmonary function assessment in this cat revealed flow limitation at the late-expiratory phase and an incomplete response to short-acting bronchodilator. The late phase of expiration in flow-volume loops predominantly reflects the function of the smaller airway, and the ratio of the peak expiratory flow (PEF) to expiratory flow at 25% of the remaining tidal volume (EF25) during tidal breathing could indicate airflow obstruction in human infants with viral bronchiolitis [21, 22]. Unlike patients with asthma, patients with bronchiolitis often showed a relatively poor or variable response to bronchodilators [4, 5, 23, 24]. As these features were also identified in our case, the ventilatory characteristics of late-expiratory flow limitation and poor reversibility to short-acting bronchodilators could raise the suspicion of bronchiolar disorders in cats.

Feline herpesvirus is prevalent in the general feline population and accounts for many cases of upper airway infection [25]. Bronchopneumonia could be caused by continuous cell-to-cell virus spread from the epithelium of the upper airways to the lower airways, occurring mainly in kittens or immunocompromised cats [26]. Nevertheless, the absence of upper airway infection could be seen in cases with pneumonia or atypical gastritis induced by herpesvirus [27, 28]. Bronchiolar epithelium is also one of the targets for feline herpesvirus [26, 29]. Viral bronchiolitis is not uncommon in human patients, and the recovery of various respiratory viruses including human herpesvirus from BAL fluid has been reported [4, 30]. However, to the authors’ knowledge, the detection of feline herpesvirus in BAL fluid has not been reported in veterinary literature. The finding in our patient suggests that viral pathogens should be investigated in cases that show uncommon imaging findings or atypical signs.

Bronchiolitis obliterans is characterized by fixed obstruction of the bronchioles with inflammation, and it can be categorized into polypoid and constrictive forms [2, 13]. The former shows the proliferation of polypoid fibroblasts within the bronchiolar lumen, whereas the later reveals concentric peribronchiolar fibrosis leading to constrictive fibroproliferative narrowing of the bronchiolar lumen. Grossly, the distribution of lesions of constrictive bronchiolitis obliterans is commonly patchy and involves more than one lung lobe [2]. These features are similar to the gross findings in our case. Furthermore, the distribution of the chronic inflammation and submucosal fibrosis was centered on bronchioles, indicating a primary bronchiolar disorder and chronic bronchiolar epithelial damage. The peribronchiolar fibrosis and inflammation that surrounds and narrows of the lumen of bronchioles in our case are consistent with constrictive bronchiolitis obliterans, and this has only been recently reported in a dog and a cat [1, 2]. However, the underlying etiology was not identified from the postmortem examination of this case. The injuries that may trigger the development of constrictive bronchiolitis obliterans include exposure to inhaled chemicals, immune-mediated disease, connective tissue disease, and preceding infection [1, 2, 13]. Owing to the lack of other explanations after extensive investigations, preceding feline herpesvirus infection was the most likely etiology considering the cat’s history, presence of herpesvirus in the BAL fluid, and lower airway neutrophilic inflammation.

An inevitable limitation for the assumption of bronchiolitis triggered by preceding feline herpesvirus infection was that the virus was not detected from the lung tissue in necropsy. Intranuclear inclusion bodies were also not found in the histopathologic specimens. Typical histopathologic findings associated with feline herpesvirus pneumonia include the presence of intranuclear inclusion bodies in sequestered bronchial and bronchiolar epithelial cells, marked neutrophil infiltration, serofibrinous exudates, extensive necrosis, and fibrinonecrotic changes in the alveolar walls [26, 27, 29]. Nevertheless, the absence of herpesvirus in the necropsied samples of this case with preceding infection is presumed possible based on several reasons. First, all feline herpesvirus cases in previous histopathological reports had developed pneumonia and mostly were young cats with acute disease process, which was in contrast to our case with a more chronic course (5 months from first presenting clinical signs to death). Second, the inclusion bodies of feline herpesvirus would disappear over time [31]. Although intranuclear inclusion bodies of feline herpesvirus were commonly noted in the previous cases [26, 27, 29], it had been reported that intranuclear inclusions were no longer found 7–10 days after inoculation in nasal mucosa in experimentally induced feline herpesvirus rhinotracheitis [31]. Third, although feline herpesvirus is prevalent in cats in our region, it has never been discovered in the BAL fluid from other cats (data not shown). Furthermore, there was no evidence suggestive of oropharyngeal contamination in the BAL sample of this cat. Therefore, it was less likely that the herpesvirus PCR in the BAL fluid was false positive due to contamination. It can be reasonably speculated that the immune-mediated reaction was triggered by feline herpesvirus to induce subsequent constrictive bronchiolitis obliterans in our cat, but the virus was no longer present at the time of necropsy as seen in chronic ocular disease initiated by feline herpesvirus [25]. However, it should be brought in mind that a preceding viral infection is a presumptive etiology rather than a definitive cause in this case.

In conclusion, the history of nonvaccinated status, lower airway neutrophilic inflammation, and presence of feline herpesvirus in the BAL fluid without coexistence of other pathogens led to the presumption that constrictive bronchiolitis obliterans was induced by a preceding feline herpesvirus infection in our case. The presence of patchy distribution of subpleural GGO on lung HRCT should raise the suspicion of peribronchiolar fibrosis. Clinical awareness of bronchiolar disorders as a differential diagnosis is important in cats with lung hyperinflation and labored breathing who show poor reversibility to bronchodilator.

## Data Availability

Data sharing is not applicable to this article as no datasets were generated or analyzed in this case report.

## Abbreviations

BAL: Bronchoalveolar lavage
BWBP: Barometric whole-body plethysmography
EF25: Expiratory flow at 25% of the remaining tidal volume
FeLV: Feline leukemia virus
FIV: Feline immunodeficiency virus
FLAD: Feline lower airway disease
GGO: Ground-glass opacities
HRCT: High-resolution computed tomography
PEF: Peak expiratory flow
pTBFVL: Pseudo-tidal breathing flow-volume loop
RI: Reference interval
